# Sacral ependymoma presents 20 years after initial posterior fossa lesion

**DOI:** 10.1136/bcr-2023-256611

**Published:** 2023-10-19

**Authors:** Lynden Guy Nicely, Mark Baxter, Sourav Banerjee, Hannah Lord

**Affiliations:** 1 Department of Cellular and Systems Medicine, School of Medicine, University of Dundee, Dundee, UK; 2 Division of Molecular and Clinical Medicine, School of Medicine, University of Dundee, Dundee, UK; 3 Tayside Cancer Centre, Ninewells Hospital and Medical School, Dundee, UK

**Keywords:** Cancer intervention, Neurooncology, Stroke, Radiology

## Abstract

Posterior fossa ependymomas (PFEs) are designated histologically as low-grade neoplasms. Despite being characterised as benign, cases of metastasis have been reported only a few times with the patients concurrently diagnosed with the primary tumour. Interval drop metastasis or spontaneous second distal tumours are extremely rare and, in most cases, are diagnosed within a few months of primary tumour resection. Here, we report a patient with a grade 2 paediatric PFE exhibiting a 20-year interval to a second sacral ependymoma. The patient was initially diagnosed with a PFE at the age of 10 years and underwent tumour resection and postoperative radiotherapy. In their late 20s, the patient presented with basilar artery occlusion complicated by life-threatening epistaxis. Post-thrombolysis, the patient presented with a large sacral grade 1 myxopapillary ependymoma with cauda equina syndrome-like symptoms. Here, we present a rare case of two ependymomas with a 20-year interval in the same patient with compounding comorbidities.

## Background

Ependymomas are low-grade gliomas and the third most common paediatric brain tumour, accounting for 2% of adult brain tumours with approximately 690 people diagnosed with spinal cord ependymoma per year in the USA.^1^ Ependymomas are usually classed as benign, encapsulated and slow-growing with associated symptoms such as headache, seizures, dizziness and nausea resulting in poor quality of life.

The WHO 2021 classification has established multiple classes of ependymomas of which posterior fossa ependymomas (PFE) and myxopapillary ependymoma (MPE) are two types. PFEs are more prevalent in children and young adults with some subtypes exhibiting a higher propensity to relapse.^2^ MPE was first reported over 90 years ago and is usually located in the cauda equina and filum terminale regions.^3^ Patients with MPE often present with pain, weakness, sensory changes, and bowel and bladder incontinence and in the vast majority of cases, drop metastatic lesions are diagnosed simultaneously with the primary tumour.

The nature of ependymomas often warrants maximal safe resection of the tumour. Postoperative radiotherapy has been shown to significantly improve event-free survival in ependymoma patients of all ages.^4 5^ Treatment results in a 5-year relative survival rate of approximately 85%.^1^ However, between 25% and 40% of survivors live with posterior fossa syndrome, a collection of neurological symptoms characterised by a reduction or absence of speech following treatment.^6^ In addition, conformal radiotherapy can induce some cognitive pathology and in some cases, cerebral vasculopathy has also been observed in paediatric patients who have undergone radiotherapy.^4 7^

Chemotherapy and targeted agents have not yet been recommended for the initial management of ependymoma in children aged over 12 months^8^ although recent evidence from the Children’s Oncology Group ACNS0831 trial shows that maintenance chemotherapy may be beneficial post-tumour debulking.^9^

In this case report, we present a patient with a large sacral mass occurring 20 years after a grade 2 PFE. Contrast CT and MRI^10–13^ scans provided a clear image of the destructive nature of the sacral mass while biopsy revealed it to be a grade 1 MPE. There have been no reported cases of a interval of 15–20 years between two distinct cases of ependymomas in the same patient.

## Case presentation

### Treatment history as a paediatric patient

The patient was diagnosed with a grade 2 PFE aged 10 years after 2–3 months history of vomiting, dizziness and blurred vision. They underwent total surgical resection of the tumour and ventriculoperitoneal shunt insertion. There were no associated symptoms of back pain or any bowel or bladder incontinence at that time. The patient was referred to radiation oncology for postoperative cranial adjuvant radiotherapy of 50 Gy in 30 fractions. This was tolerated well initially but there were subsequent cognitive deficits likely due to late effects of radiation treatment.

### Later treatment history as an adult patient

The patient remained well until their late 20s when they experienced a sudden decline in neurological function with left-sided weakness and dysphasia. This was found to be due to a basilar artery thrombus. The patient received thrombolysis, complicated by life-threatening epistaxis. Thereafter, they made a slow but steady recovery over 12 months and were able to walk again, although not independently and speech improved to normal.

Two years later, the patient presented to the hospital with worsening mobility and falls. A consequent neurological assessment was carried out for the patient. The patient was in a wheelchair but was able to mobilise from the chair to the couch. Power in upper limbs was 5/5, while power in lower limbs was 4/5. Reduced coordination was observed in the lower limbs but reflexes were brisk in the lower limbs while post pointing and disdiadochokinesis were observed in the upper limbs. Marked nystagmus was observed on the left and right lateral gaze.

The patient also developed sphincter disturbance requiring catheterisation and low back pain requiring opiate analgesia. These symptoms were attributed to sequelae of the basilary artery thrombus and further imaging was not initially performed. As the condition progressed, the patient was wheelchair users and had increased lower limb weakness and fever that was initially attributed to discitis.

## Investigations

**Figure 1:**
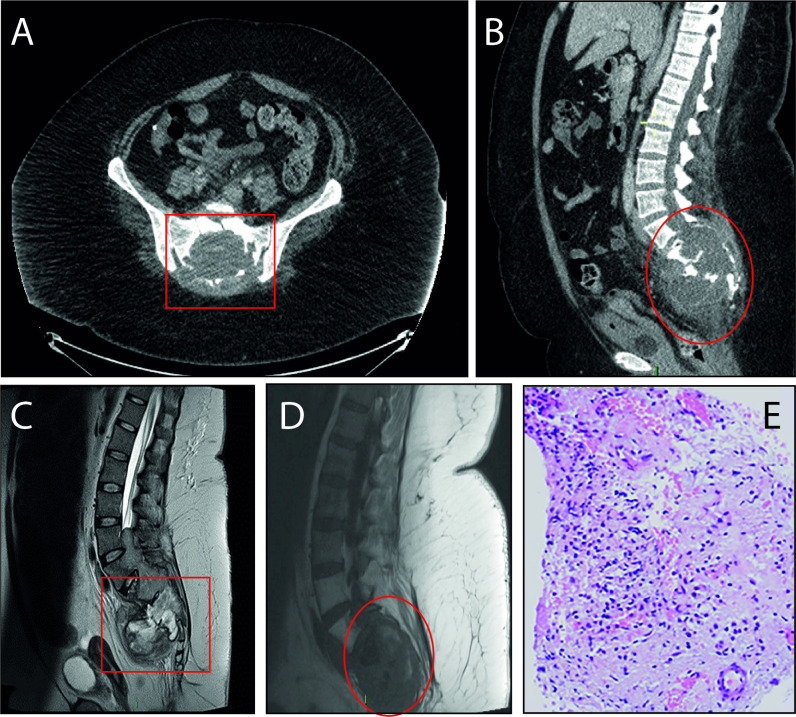
Patient imaging and pathology of the tumour. (A, B) Contrast CT scan of the abdomen and pelvis along with (C, D) sagittal T2 and T1 MRI showing the destruction of the sacrum by a large, predominately cystic slow mass, indicative of a myxopapillary ependymoma. There is bilateral extradural extension into the piriformis muscles and there is no involvement of the sciatic nerves. Red box/ellipses indicate the sacral mass. (E) Histopathology image suggestive of an infarcted tumour and viable regions positive of glial fibrillar acidic protein and epithelial membrane antigen. Secondary reactive changes are evident along with normal appearing skeletal muscle fibres. The appearances are thought to represent a glial tumour, most likely myxopapillary ependymoma WHO grade 1.

A contrast CT scan of the abdomen and pelvis was performed in view of the severity of pain (figure 1A,B), which showed a large soft tissue mass at the sacrum, with a multiloculated fluid component, rim enhancing partially calcified walls, with little inflammatory change. There was an impression of perineural spread along the sciatic nerves.

An MRI scan (figure 1C,D) showed a predominately cystic slow growing mass centred on the distal spinal canal. There was gross abnormality of the lower spine with a predominantly intradural 12 cm (craniocaudal) by 9 cm (transverse) mass. The superior margin lay at the level of the L3–L4 intervertebral disc, and its caudal margin was at S3. The mass was predominantly cystic but anteriorly and posteriorly there were solid components.

On postcontrast scans, the solid components and the cyst walls underwent avid contrast enhancement. The mass had caused bony remodelling and scalloping of adjacent vertebral bodies and widening of the spinal canal. Centrally, the mass contained low signal foci and these corresponded to bone fragments depicted on the CT scan (figure 1A,B). There was evidence of bilateral extradural extension into the piriformis muscles with no involvement of the sciatic nerves (lying anteriorly). The cervical and thoracic cord were normal. No evidence of proximal metastases was observed. There had been previous surgery to the fourth ventricle and cerebellar vermis, but no other abnormalities were observed elsewhere in the imaged spine or brain.

A positron emission tomography scan showed increased tracer uptake along with the peripheral aspects of the tumour (Standardized Uptake Value SUV max 13.9 g/mL) and central photopenia. Initially, the possibility of a sacral chordoma was raised. This was discussed at the National Chordoma meeting and a wider differential was proposed. In view of diagnostic uncertainty, a biopsy was performed. Histology confirmed an MPE grade 1 (figure 1E) with glial fibrillar acidic protein and epithelial membrane antigen positivity suggestive of glial origin.

## Differential diagnosis

Prior to the biopsy confirmation, there were a range of differential diagnoses considered. These included an MPE, chordoma, metastasis from a primary malignancy located elsewhere, or less likely, a sarcoma or lymphoma. Ependymoma was felt to be the most likely and this was subsequently confirmed.

Recurrent ependymoma after more than 20 years are uncommon^14^ and the median time to first recurrence has been observed as 16 months.^15^ It is also rare for grade 1 ependymoma to be destructive of bony tissue. Drop metastases are widely described,^3^ but a 20-year interval was considered unlikely. The second tumour could possibly be a spontaneous tumour independent of the PFE.

## Treatment

Following discussion at the neuro-oncology multidisciplinary team review and with neuro-oncologists at a national level, surgical resection of the sacral ependymoma was ruled out due to the highly destructive nature of the tumour as well as its location in the sacral spine and proximity to the cauda equina. The patient received palliative radiotherapy using 50 Gy in 28 fractions in a three-dimensional conformal plan, with once-daily treatments.

## Follow-up and outcome

At first review postradiotherapy for the tumour, the patient had returned to mobilising with a tri-wheeler, no longer requiring a wheelchair, in most part because her pain control was significantly better. Opiate requirements were less, and a follow-up CT showed a reduction in tumour dimensions from 9.5 cm×7.5 cm×12.5 cm to 7.2 cm×4.8 cm×7.6 cm.

A few months following this, the patient was admitted to hospital with a ventriculoperitoneal shunt infection after a 2-week history of lethargy and progressive reduction in responsiveness. Over the course of 12 days, their condition deteriorated despite broad-spectrum antibiotics. In the absence of clear reversible and treatable aetiology, treatment was withdrawn and anticipatory care medications were prescribed. The cause of death was recorded as ventriculoperitoneal shunt associated meningitis with cerebrovascular disease as a contributing factor.

## Discussion

Here, we present the unique case of a grade 1 sacral MPE in adulthood following a childhood posterior fossa grade 2 ependymoma in a patient with significant functional impairment.

A previous case report described a diagnosis of a drop metastasis in the sacral area 19 months after an MPE, but it was of the same grade and hence classed as recurrence.^16^ Our case presents a diagnosis after 20 years, a previously unreported time interval. To date, no literature reports drop metastasis of a lower grade of ependymoma from a higher-grade lesion of distinct subtype. Therefore, we suggest the sacral MPE in our case is most likely a spontaneous tumour arising independent of the paediatric PFE.

Guidelines for recurrence are for gross total resection with adjuvant radiotherapy^17^ to improve local control and long-term survival. Due to the extent of the disease in this case, resection was not feasible and only a biopsy was performed to confirm diagnosis. In our case, radiotherapy was given for palliative reasons to help with pain control and minimise further local destruction.

The patient was treated with adjuvant radiotherapy post resection of their original PFE. This is standard care and deemed safe after extensive longitudinal studies by multiple groups.^4 8^ However, a few statistical outliers are observed where some paediatric patients developed radiotherapy-like-toxicity symptoms including cognitive deficits and in rare cases stroke and coma.^18^ The current patient did suffer from cognitive deficits and eventually a stroke in adulthood which begs the question as to whether adjuvant radiotherapy should always be used in all paediatric PFE patients, or whether some stratification is required to determine fitness. Late toxicity from radiotherapy is poorly understood in terms of the mechanisms of neurotoxicity and neurodegeneration.^19^ This highlights the importance of monitoring for long-term sequelae in survivors of ependymoma, particularly in the context of childhood cancer survivors.

In this case, the patient developed paraesthesia at the age of 27 in their lower limbs. The possibility of a second tumour was not considered, with the symptoms being attributed to the stroke. Therefore, further investigation was not performed at that stage. These symptoms, in all likelihood, could have been caused by the sacral MPE, which was subsequently diagnosed 4 years later.

This case highlights the delay in diagnosis, the potential toxicities associated with radiotherapy, and the unusually aggressive nature of a rare second ependymoma opening up speculations of radiation toxicity, 20-year interval distal drop metastasis or delayed spontaneous secondary tumour within the same patient.

Hence, it is critical to consider late treatment toxicity, secondary tumours of differing pathology and the importance of monitoring for long-term sequelae in survivors of ependymoma, particularly in the context of childhood cancer survivors.

**Patient’s perspective**

‘Mum tells me that it was difficult to get the initial diagnosis of my first tumour when I was a child. It took a very long time to be investigated and to be taken seriously. At one stage, my mum was so distressed that 2 security guards escorted her from the hospital. All of this must have been very emotional for her. When I eventually got the diagnosis, it was very scary for my family. Cancer at that age and stage is terrifying, but it was good to finally be taken seriously. I’m glad my mum followed her instinct.’

‘It was strange to think that the tumour on my spine had been sitting there on my spine for so long. I had a sore back but didn’t think anything of it. My general practitioner (GP) blamed the sore back on my weight.’

‘When I had my stroke, I didn’t want to know anything about my condition. It was a lot to take in but I have been taking things step by step. I have good support and carers to help me out. I often go round to dinner with my mum. My pain is also being managed much better than it was for quite some time after the stroke.’

‘My experience of medical professionals have been mixed, from exceptionally kind to those who would not believe me or my family. My journey has been frightening for everyone at every stage. I am proud of myself with where I have got to. I have far exceeded everyone’s expectations of me after my stroke. Today, I live independently from my mum and I am well supported by her and carers.’

**Learning points**

- This is a unique case reporting a second tumour of a differing grade of ependymoma diagnosed after a 20-year interval.
- Potential toxicity from adjuvant radiotherapy should be considered in all patients, especially those irradiated in childhood.
- Greater clinical curiosity should be employed when investigating complications in patients with a clinical history of cancer.
